# Supplementary figures and images for: The role of TAp63γ and P53 point mutations in regulating DNA repair, mutational susceptibility and invasion of bladder cancer cells
Source: eLife. 2021 Nov 8;10:e71184. doi: 10.7554/eLife.71184 (PMC8575459; doi:10.7554/eLife.71184)

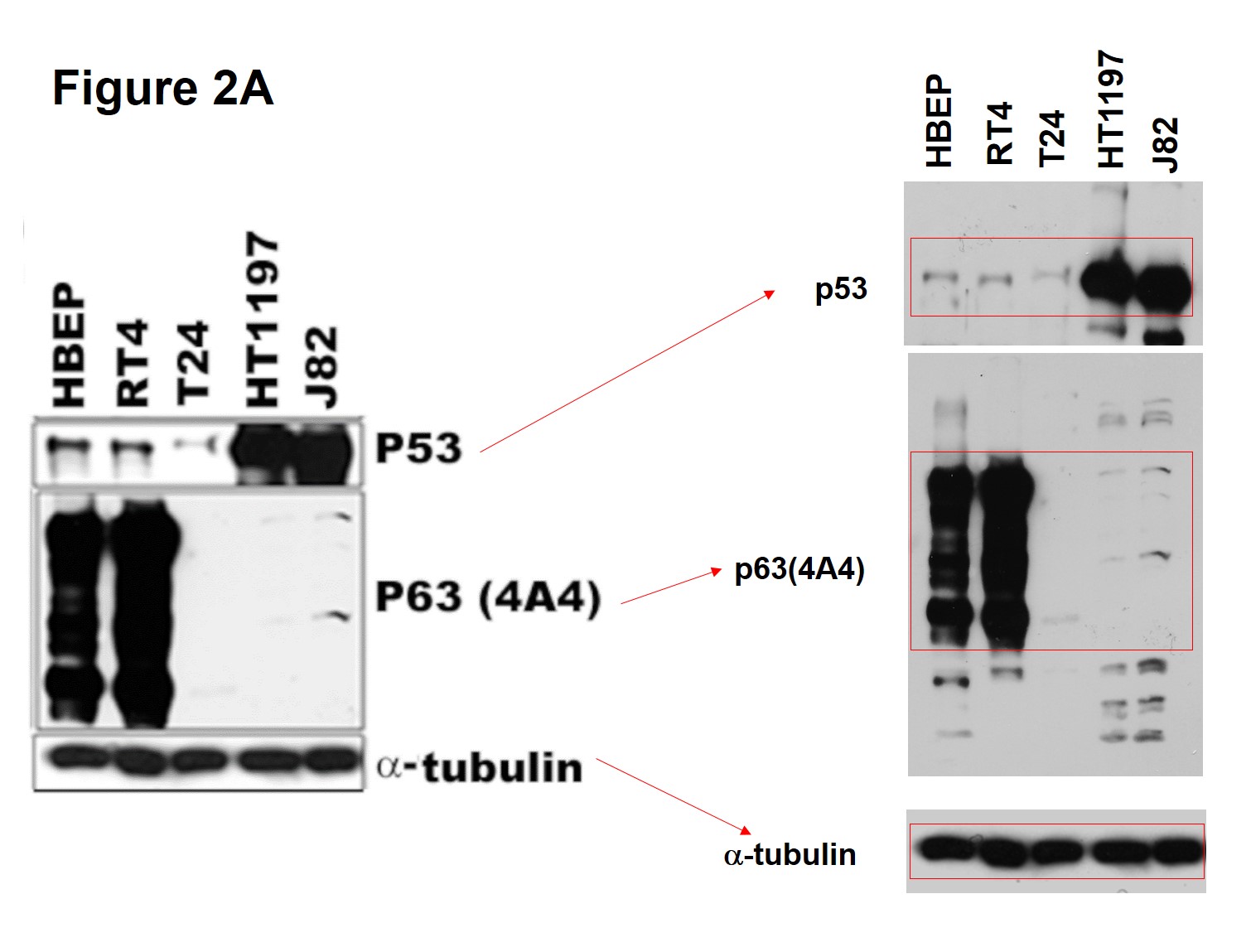

Supplement: Figure 2—source data 1. [file elife-71184-fig2-data1.zip › Figure 2A-Source data.jpg]

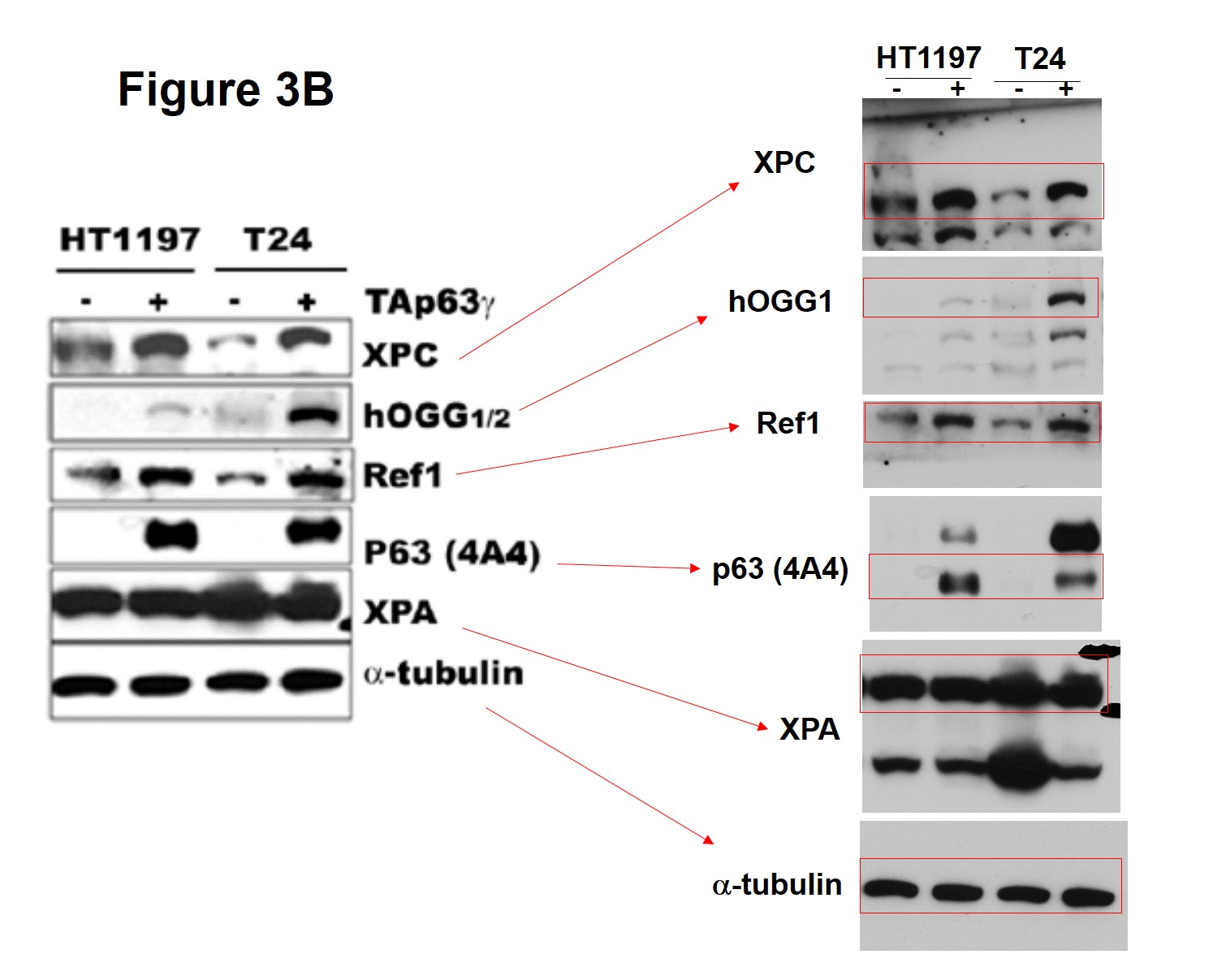

Supplement: Figure 3—source data 1. [file elife-71184-fig3-data1.zip › Figure 3B-Source data.jpg]

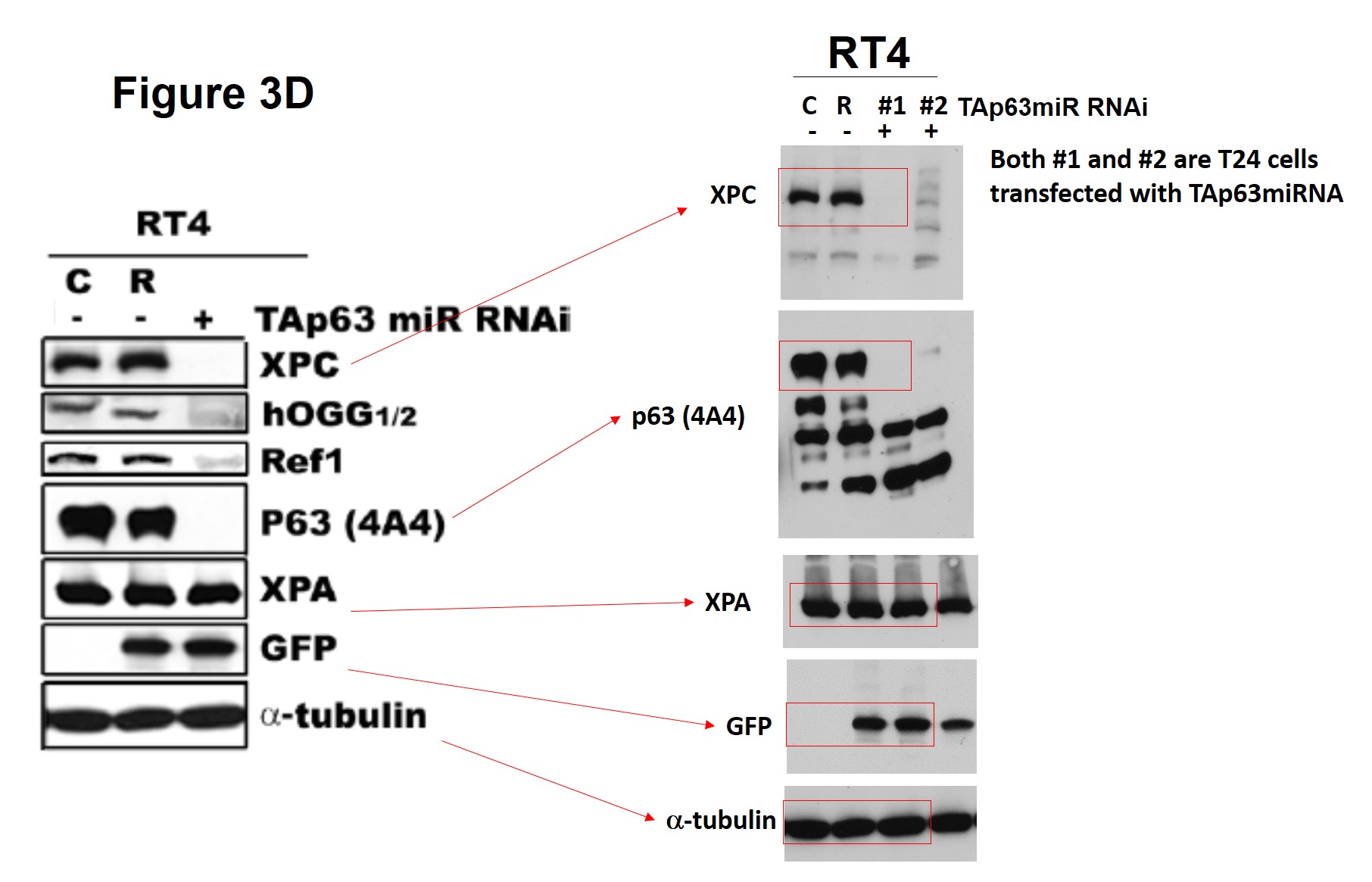

Supplement: Figure 3—source data 2. [file elife-71184-fig3-data2.zip › Figure 3D-Source data.jpg]

# BTT

**KDa** 70 -  
55 -

|  | Ta |    | T1 |    | T2 |    | T3/T4 |    | Stage |
|--|----|----|----|----|----|----|-------|----|-------|
|  | 8  | 54 | 55 | 56 | 26 | 31 | 73    | 61 | 10 62 |

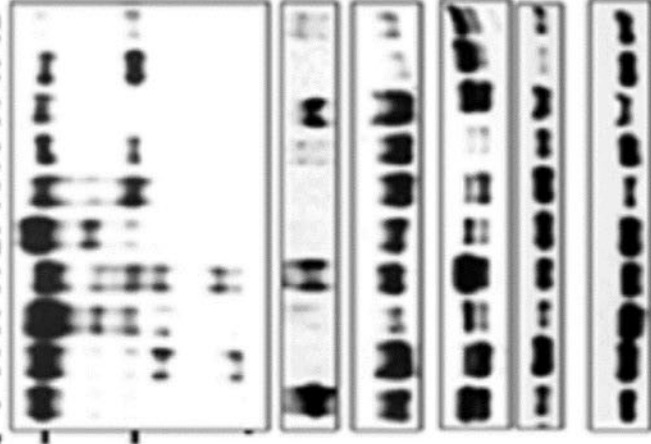

p63 (4A4)

XPC

XPA

hOGG<sub>1/2</sub>

Ref1

α-tubulin

Ta T1 T2 T3/T4

RT4 1 33 8 54 55 56 26 31 73 61 10 62 63

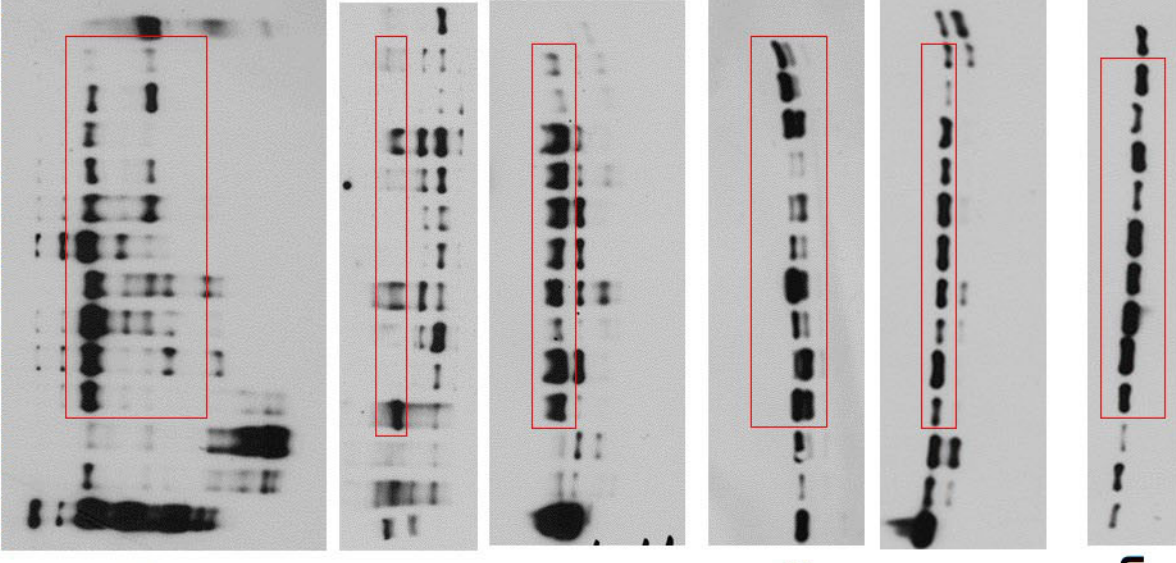

Supplement: Figure 5—source data 1. [file elife-71184-fig5-data1.pdf]
