# Supplementary material for: The role of TAp63γ and P53 point mutations in regulating DNA repair, mutational susceptibility and invasion of bladder cancer cells
Source: eLife. 2021 Nov 8;10:e71184. doi: 10.7554/eLife.71184 (PMC8575459; doi:10.7554/eLife.71184)
Supplement: Supplementary file 1. [file elife-71184-supp1.docx]

**Supplementary File 1**

**UV- and H_2_O_2_-DNA damage^1^ induce more mutations^2^ in MIBC (T24 & HT1197) than NMIBC (RT4) cells and normal human urothelial (UROtsa) cells.**

| Cell type | Treatment | | | Exp. | Mutant colonies /  Total colonies | Mutation Frequency (X10^4^) | | Fold Change | | P-value |  |
| --- | --- | --- | --- | --- | --- | --- | --- | --- | --- | --- | --- |
| UROtsa | | Control | 1 | | 7/24889 | | 2.8 | | 1.0 |  | |
|  |  | UV | 1 | | 61/11423 | | 53.4 | | 19.1 |  | |
|  |  | H_2_O_2_ | 1 | | 73/12997 | | 56.1 | | 20 |  | |
| RT4 | | Control | 1 | | 3/12242 | | 2.5 | | 1.0 |  | |
|  |  | UV | 1 | | 17/4273 | | 39.8 | | 16.2 |  | |
|  |  | H_2_O_2_ | 1 | | 11/2193 | | 50.2 | | 20.5 |  | |
| T24 | | Control | 1 | | 5/14915 | | 3.4 | | 1.0 |  | |
|  |  |  | 2 | | 6/14016 | | 4.3 | | 1.0 |  | |
|  |  | UV | 1 | | 119/2698 | | 441.1 | | 131.7 | 0.00389 | |
|  |  |  | 2 | | 161/3186 | | 505.3 | | 118.1 |  | |
|  |  | H_2_O_2_ | 1 | | 198/5266 | | 376 | | 112.2 | 0.00024 | |
|  |  |  | 2 | | 180/3720 | | 483.9 | | 113.1 |  | |
| HT1197 | | Control | 1 | | 9/35554 | | 2.5 | | 1.0 |  | |
|  |  |  | 2 | | 8/22646 | | 3.5 | | 1.0 |  | |
|  |  | UV | 1 | | 108/12104 | | 89.2 | | 35.3 | 0.00201 | |
|  |  |  | 2 | | 119/9096 | | 130.8 | | 37.1 |  | |
|  |  | H_2_O_2_ | 1 | | 136/3343 | | 406.8 | | 160.8 | 0.00615 | |
|  |  |  | 2 | | 142/3092 | | 495.3 | | 140.3 |  | |

^1^ Plasmid pSB189 DNAs which contain the *supF* gene were irradiated with UVC (1500 J/m^2^) or modified with H_2_O_2_ (100 mM, 1 h at 37 ^o^C).

^2^ Mutations in the *supF* gene were detected as in **Fig. 1**.
