# Supplementary material for: The role of TAp63γ and P53 point mutations in regulating DNA repair, mutational susceptibility and invasion of bladder cancer cells
Source: eLife. 2021 Nov 8;10:e71184. doi: 10.7554/eLife.71184 (PMC8575459; doi:10.7554/eLife.71184)
Supplement: Supplementary file 2. [file elife-71184-supp2.docx]

**Supplementary File 2**

**Enforced TAp63γ expression^1^ reduces UV- and H_2_O_2_-DNA damage^2^ induced mutations^3^ in MIBC (T24 & HT1197) cells.**

| Cell Type |  | Stable Transfectant | Treatment | Mutant colonies /  Total colonies | | Mutation Frequency (X10^4^) | |
| --- | --- | --- | --- | --- | --- | --- | --- |
| T24 | Vector | | Control | 6/17424 | 3.4 | |  |
|  |  |  | UV | 370/9440 | 391.9 | |  |
|  |  |  | H_2_O_2_ | 160/5040 | 317.5 | |  |
|  | TAp63γ | | Control | 12/38560 | 3.1 | |  |
|  |  |  | UV | 310/25120 | 123.4 | |  |
|  |  |  | H_2_O_2_ | 550/37120 | 148.2 | |  |
| HT1197 | Vector | | Control | 10/26220 | 3.6 | |  |
|  |  |  | UV | 368/23184 | 158.7 | |  |
|  |  |  | H_2_O_2_ | 360/8820 | 408.2 | |  |
|  | TAp63γ | | Control | 15/47350 | 3.2 | |  |
|  |  |  | UV | 143/15352 | 92.8 | |  |
|  |  |  | H_2_O_2_ | 239/10488 | 228 | |  |

^1^ The stable transfectants were constructed as described in **Fig. 2**.

^2^ Plasmid pSB189 DNAs which contain the *supF* gene were irradiated with UVC (1500 J/m^2^) or modified with H_2_O_2_ (100 mM, 1 h at 37 ^o^C).

^3^ Mutations in the *supF* gene were detected as in **Fig. 1**.
