# Supplementary material for: The role of TAp63γ and P53 point mutations in regulating DNA repair, mutational susceptibility and invasion of bladder cancer cells
Source: eLife. 2021 Nov 8;10:e71184. doi: 10.7554/eLife.71184 (PMC8575459; doi:10.7554/eLife.71184)
Supplement: Supplementary file 3. [file elife-71184-supp3.docx]

**Supplementary File 3**

**Knockdown TAp63γ expression^1^ enhances UV- and H_2_O_2_-DNA damage^2^ induced mutations^3^ in NMIBC (RT4) cells.**

| Cell Type | Treatment | Mutant colonies /  Total colonies | Mutation Frequency (X10^4^) | |
| --- | --- | --- | --- | --- |
| RT4 | Control | 2/9521 | 2.1 |  |
|  | UV | 20/5293 | 37.8 |  |
|  | H_2_O_2_ | 25/5231 | 47.8 |  |
| RT4 TAp63 miRNAi | Control | 10/36352 | 2.8 |  |
|  | UV | 148/9196 | 160.9 |  |
|  | H_2_O_2_ | 106/8352 | 126.9 |  |

^1^ The stable transfectants were constructed as described in **Fig. 2.**

^2^ Plasmid pSB189 DNAs which contain the *supF* gene were irradiated with UVC (1500 J/m^2^) or modified with H_2_O_2_ (100 mM, 1 h at 37 ^o^C).

^3^ Mutations in the *supF* gene were detected as in **Fig. 1**.
