# Supplementary material for: The role of TAp63γ and P53 point mutations in regulating DNA repair, mutational susceptibility and invasion of bladder cancer cells
Source: eLife. 2021 Nov 8;10:e71184. doi: 10.7554/eLife.71184 (PMC8575459; doi:10.7554/eLife.71184)
Supplement: Transparent reporting form [file elife-71184-transrepform1.docx]

***eLife’s* transparent reporting form**

We encourage authors to provide detailed information *within their submission* to facilitate the interpretation and replication of experiments. Authors can upload supporting documentation to indicate the use of appropriate reporting guidelines for health-related research (see [EQUATOR Network](http://www.equator-network.org/%20)), life science research (see the [BioSharing Information Resource](https://biosharing.org/)), or the [ARRIVE guidelines](http://www.plosbiology.org/article/info:doi/10.1371/journal.pbio.1000412) for reporting work involving animal research. Where applicable, authors should refer to any relevant reporting standards documents in this form.

If you have any questions, please consult our Journal Policies and/or contact us: [editorial@elifesciences.org](mailto:editorial@elifesciences.org).

**Sample-size estimation**

- You should state whether an appropriate sample size was computed when the study was being designed
- You should state the statistical method of sample size computation and any required assumptions
- If no explicit power analysis was used, you should describe how you decided what sample (replicate) size (number) to use

Please outline where this information can be found within the submission (e.g., sections or figure legends), or explain why this information doesn’t apply to your submission:

Fig.1A- The experiments have been repeated at least twice to obtain the mean value for mutation frequency. Student t-test has been used to calculate p-value as well as the standard deviation (STDEV). The statistic calculations are presented in Supplementary file1-3.

Fig.1B &1C: The experiments have been conducted to measure global DNA repair activity from multiple cell lines. Data are calculated from results of multiple time points.

Fig. 1E & 1F: The experiments have been conducted to measure the DNA-damage-dependent- repair synthesis activity from several cell lines. Two different DNA damaging agents (UV and H_2_O_2_) were used for inducing DNA damage. Experiments have been conducted twice.

Fig.1D- The experiments have been conducted to measure transcription-dependent (transcription-coupled repair) DNA repair activity from 4 different cell lines. Two different DNA damaging agents (UV and H_2_O_2_) were used for inducing DNA damage. Data are calculated from results of multiple UV doses and H_2_O_2_concentrations. Experiments have been conducted twice.

Fig. 2A & 2B & 2C & 2E- The experiments have been done to measure protein expression and DNA repair activity from multiple cell lines.

Fig. 2D & 2F: The experiments have been conducted to obtain the mean value for mutation frequency. This type of experiments has been performed multiple times for calculating statistic significance (Supplementary file 1-3).

Fig. 3A & 3B & 3C & 3E- The experiments have been done to measure mRNA and protein expression from multiple cell lines. The experiments have been conducted multiple times.

For Figure-4A:

A total 5x10^5^ cells/2.5 ml/well per control or different transfectants were triplicated applied to the migration and invasion chambers following the vendor’s instruction. The experiments have been repeated at least twice to obtain the mean value for % of migration and % of invasion as well as the standard deviation (STDEV).

For figure 4B:

A total 1x10^6^ cells/2.5 ml/well per control or different transfectants were duplicated applied to a 6-well plate. At least twice experiments per cell group have been conducted to determine the motility activity.

The same description can also be found in the section of material and methods under Cell motility assay.

Fig. 5A & 5C: The experiments have been done to measure DNA damage dependent repair synthesis activity and protein expression from multiple clinical samples (n=16). Two different DNA damaging agents (UV and H_2_O_2_) were used for inducing DNA damage.

**Replicates**

- You should report how often each experiment was performed
- You should include a definition of biological versus technical replication
- The data obtained should be provided and sufficient information should be provided to indicate the number of independent biological and/or technical replicates
- If you encountered any outliers, you should describe how these were handled
- Criteria for exclusion/inclusion of data should be clearly stated
- High-throughput sequence data should be uploaded before submission, with a private link for reviewers provided (these are available from both GEO and ArrayExpress)

Please outline where this information can be found within the submission (e.g., sections or figure legends), or explain why this information doesn’t apply to your submission:

Fig.1A & D- The experiments have been repeated at least twice to obtain the mean value for mutation frequency and have been used student t-test to get p-value as well as the standard deviation (STDEV).

Fig. 1B &1C &1E: The experiments have been done NER and BER from same samples.

Fig. 2B & 2C & 2E- The experiments have been done NER and BER from same samples.

Fig. 2D & 2F: The experiments have been repeated at least twice.

Fig. 3A & 3B & 3C & 3E- The experiments have been done RT-PCR and Western blot from same samples.

For Figure-4A:

A fixed amount of either control or different transfectants were triplicated applied to the migration and invasion chambers following the vendor’s instruction. The experiments have been repeated at least twice to obtain the mean value for % of migration and % of invasion as well as the standard deviation (STDEV).

For figure 4B:

Each cell group was duplicated applied to a 6-well plate. At least twice experiments per cell group have been conducted.

The same description can also be found in the section of material and methods under Cell motility assay.

Fig. 5A: The experiments have been done NER and BER from same samples.

Fig. 5C: The experiments have been done once due to lack of amounts of clinical samples.

**Statistical reporting**

- Statistical analysis methods should be described and justified
- Raw data should be presented in figures whenever informative to do so (typically when N per group is less than 10)
- For each experiment, you should identify the statistical tests used, exact values of N, definitions of center, methods of multiple test correction, and dispersion and precision measures (e.g., mean, median, SD, SEM, confidence intervals; and, for the major substantive results, a measure of effect size (e.g., Pearson's r, Cohen's d)
- Report exact p-values wherever possible alongside the summary statistics and 95% confidence intervals. These should be reported for all key questions and not only when the p-value is less than 0.05.

Please outline where this information can be found within the submission (e.g., sections or figure legends), or explain why this information doesn’t apply to your submission:

Fig.1A & D- The experiments have been repeated at least twice to obtain the mean value for mutation frequency and have been used student t-test to get p-value as well as the standard deviation (STDEV).

Fig. 1B &1C &1E: Not applicable

Fig. 2A & 2B & 2C & 2E: Not applicable

Fig. 2D & 2F: The experiments have been repeated at least twice to obtain the mean value for mutation frequency and have been used student t-test to get p-value as well as the standard deviation (STDEV).

Fig. 3A & 3B & 3C & 3E- Not applicable

For figure 4A:

Each cell group was triplicated applied to the migration and invasion chambers. At least twice experiments per cell group have been conducted. The mean values per cell group for RM and RI were determined from the 6 times cell counting results. The STDEV was determined accordingly.

For figure 4B: Not applicable

Fig. 5A & 5C: Not applicable

(For large datasets, or papers with a very large number of statistical tests, you may upload a single table file with tests, Ns, etc., with reference to sections in the manuscript.)

**Group allocation**

- Indicate how samples were allocated into experimental groups (in the case of clinical studies, please specify allocation to treatment method); if randomization was used, please also state if restricted randomization was applied
- Indicate if masking was used during group allocation, data collection and/or data analysis

Please outline where this information can be found within the submission (e.g., sections or figure legends), or explain why this information doesn’t apply to your submission:

Fig.1- We mentioned in the figure legend.

Fig.2- We mentioned in the figure legend.

Fig.3- We mentioned in the figure legend.

For figure 4:

The different transfectant was side by side compared with either control or vector control group.

Fig.5- We mentioned in the figure legend.

**Additional data files (“source data”)**

- We encourage you to upload relevant additional data files, such as numerical data that are represented as a graph in a figure, or as a summary table
- Where provided, these should be in the most useful format, and they can be uploaded as “Source data” files linked to a main figure or table
- Include model definition files including the full list of parameters used
- Include code used for data analysis (e.g., R, MatLab)
- Avoid stating that data files are “available upon request”

Please indicate the figures or tables for which source data files have been provided:

The original images are uploaded as requested.

The original images as represented per cell group are shown in Figure 4.
